# Retrospective US database analysis of persistence with glatiramer acetate vs. available disease-modifying therapies for multiple sclerosis: 2001–2010

**DOI:** 10.1186/1471-2377-14-11

**Published:** 2014-01-14

**Authors:** MerriKay Oleen-Burkey, Anissa Cyhaniuk, Eric Swallow

**Affiliations:** 1 Outcomes Scribe, LLC, 664 Wynding Oaks, Kalamazoo, MI 49006, USA; 2 Formerly, Health Economics and Outcomes Research, Teva Pharmaceuticals, 901 E. 104th Street, Suite 900, Kansas City, MO 64131, USA; 3 OptumInsight Life Sciences, 13625 Technology Drive, Eden Prairie, MN 55344, USA

**Keywords:** Multiple sclerosis, Disease-modifying therapy, Persistence, Treatment gaps, Therapy re-initiaton, Therapy switching, Therapy discontinuation

## Abstract

**Background:**

Long-term persistence to treatment for chronic disease is difficult for patients to achieve, regardless of the disease or medication being used. The objective of this investigation was to examine treatment persistence with glatiramer acetate (GA) relative to available disease-modifying therapies (DMT) for multiple sclerosis (MS) over 12-, 24- and 36-month periods.

**Methods:**

Data from Clinformatics™ for DataMart affiliated with OptumInsight was used to identify patients using DMT between 2001 and 2010. Patients with 12, 24, and 36 months of follow-up were included. Persistence was defined as continuous use of the same DMT for the duration of follow-up regardless of treatment gaps. Regimen changes including re-initiation of therapy following gaps of 15 days or more, switching therapy, and DMT discontinuation were investigated. Descriptive statistics were used to summarize the results.

**Results:**

Cohorts of GA users with 12 months (n = 12,144), 24 months (n = 7,386) and 36 months (n = 4,693) of follow-up were identified. Persistence rates with GA were 80% for all time periods; discontinuation rates declined over time while switching increased modestly. In contrast, the full DMT-treated cohorts showed persistent rates of 68.3% at 12 months (n = 35,312), 53.9% at 24 months (n = 21,927), and 70.1% at 36 months (n = 14,343). As with these full DMT-treated cohorts, the proportion of GA users remaining on their initial therapy without a gap of 15 days or more decreased with length of follow-up. However, the proportion of GA users with a gap in treatment who re-initiated GA increased over time (64.4% at 12 months; 75.1% at 24 months, and 80.1% at 36 months) while those in the full DMT-treated cohorts re-initiated therapy at rates of only 50-60%.

**Conclusions:**

Persistence rates for GA were 80% for the 12-, 24- and 36-month time periods in contrast with the full DMT-treated cohorts whose persistence rates never exceeded 70.0%. Although there were more gaps in therapy of 15 days or more with all DMT over time, the proportion of GA users re-initiating therapy increased with follow-up contributing to the steady persistence. Therapy persistence is essential to achieve the desired outcomes in MS.

## Background

Multiple sclerosis (MS) is a neurological condition characterized by axonal loss and acute focal inflammatory demyelination [1]. The incidence of MS in the white population of the United States (US) in 2000 was estimated at 7.3 cases per 100,000 individuals, and the prevalence rate was 191 cases per 100,000 individuals [2]. The disease affects approximately 400,000 in the US and 2.3 million people globally [3]. Diagnosis typically occurs between 20 and 50 years of age [3]. MS occurs more frequently in women than men and in Caucasians more often than in other ethnic groups [4]. Approximately 85% of the cases of MS are initially diagnosed as relapsing-remitting multiple sclerosis (RRMS), which is characterized by disease exacerbations (new or recurring symptoms) and periods of remission [5].

Disease-modifying therapies (DMT) in conjunction with usual symptomatic and supportive care are the primary treatments for MS. Currently available DMTs include glatiramer acetate (GA; Copaxone, Teva Pharmaceuticals USA, Inc., North Wales, PA), intramuscular interferon beta-1a (IFNβ-1a IM; Avonex, Biogen Idec, Cambridge, MA), subcutaneous interferon beta-1a (IFNβ-1a SC; Rebif, EMD Serono Inc., Rockland, MA), interferon beta-1b (IFNβ-1b; Betaseron, Bayer Healthcare Pharmaceuticals, Inc., Montville, NJ; Extavia; Novartis Pharmaceutical Corporation, East Hanover, NJ), natalizumab (NZ; Tysabri, Biogen Idec, Cambridge, MA), fingolimod (FG; Gilenya, Novartis Pharmaceutical Corporation, East Hanover, NJ), teriflunomide (TF; Aubagio, Genzyme Corporation, Cambridge, MA) and dimethyl fumarate (DF; Tecfidera, Biogen Idec, Cambridge, MA). Although they are not curative, DMTs have been shown to reduce the occurrence of relapses and slow progression of disability [6-13].

Similar to patients with other chronic diseases, MS patients often have gaps in treatment, sometimes switch therapies, or discontinue treatment altogether. In a chart review of MS patients, treatment interruptions were most frequent in the first 6 months after initiation, with perceived lack of efficacy the most common reason for interruption [14]. In other studies, key factors identified as influencing discontinuation and persistence failure in MS patients have included adverse events, fatigue associated with MS treatment, lack of efficacy, injection anxiety/problems, and patient out-of-pocket cost [15-22]. A study of patients at a large, multispecialty physician practice in the US also showed the early decline in DMT persistence. Of 25 patients initiating a DMT in the two-year study period, 26% were non-persistent to therapy within 4 months, 36% within 8 months and 42% within 14 months after initiation [23].

Although the published literature includes several studies dealing with persistence to DMT, most have involved small samples of patients with MS and did not follow them to look for persistence trends over three years or more. The objective of this study is to investigate treatment persistence with GA relative to available DMT for MS in the 2001 to 2010 time frame over 12-, 24-, and 36-month periods.

## Methods

### Data source

The administrative patient claims data used in this study included pharmacy claims, and patient eligibility information along with medical claims from United Health Group (UHG) and non-UHG plans. The individuals covered by these health plans, about 32 million annual lives in 2010, are geographically diverse across the US, with greatest representation in the South and Midwest US census regions. The plans provide fully insured coverage for outpatient prescription medication, professional (e.g., physician), and facility (e.g., hospital) services. Outpatient pharmacy claims provide, among other information, National Drug Codes (NDC) for dispensed medications, quantity dispensed, drug strength, days supply, provider specialty code, and health plan and patient costs.

No identifiable protected health information was extracted or accessed during the course of the study. Pursuant to the Health Insurance Portability and Accountability Act, the use of de-identified data does not require Institutional Review Board approval or waiver of authorization [24].

### Patient identification

Patients were selected for this analysis if they had evidence of MS as shown by an International Classification of Diseases, Ninth Revision (ICD-9) diagnostic code 340 and a claim for a marketed MS therapy between January 2001 and December 2008. Patients were required to have at least one claim for a marketed DMT during this time period as well as at least 24 months of insurance eligibility from the first treatment identified during the analysis period; this extended the analysis window to December 2010. MS therapies included in this analysis were GA, IFNβ-1a IM, IFNβ-1a SC, IFNβ-1b, and NZ. The oral therapies, FG, TF and DF, were not included as they either entered the market near the end of this analysis period (FG) and users did not meet the inclusion criteria, or they have come to the market since 2010 (TF and DF).

### Subject cohorts

Three patient cohorts were examined based on follow-up information: patients with a minimum of 12 months of follow-up from the time of initiating a DMT, patients with a minimum of 24 months of follow-up, and patients with a minimum of 36 months of follow-up. These cohorts were not mutually exclusive; i.e., patients in the 36-month group could also be included in the 12 and 24-month groups. *Persistence* was defined as continuous use of the same DMT for the duration of follow-up regardless of treatment gaps. Analyses of persistence and regimen changes were developed for patients initiating GA therapy and the cohort of those initiating any available DMT in the time frame.

### Regimen changes

A patient was defined as having a *gap in therapy* if a prescription was not filled for the initial DMT before the days supply of its previous prescription plus a 15-day grace period expired. Fifteen days was used in this definition based on an analysis of the entire ten years of claims data that revealed that this was the most common threshold for a therapy gap. A patient was defined as having *re-initiated therapy* if s/he had a gap in initial DMT of at least 15 days and then filled another prescription for the same therapy at some point after the gap. A *switch in therapy* was defined as a prescription fill for a marketed MS therapy other than the initial DMT in a case where the patient stopped filling prescriptions for the initial MS therapy. *Combination therapy* was defined as a prescription fill for a marketed MS therapy other than the initial DMT prior to a gap occurring in the initial DMT; it also required a subsequent prescription fill for the initial DMT to confirm continued use. Patients were double counted if they had more than one regimen change in the analysis period. As a result, the total percentages presented below may be greater than 100%.

## Results

### Sample characteristics

There were 35,312 MS patients with 12 months of follow-up including 12,144 (34.4%) patients on GA therapy; 21,927 patients were identified with 24 months of follow-up, including 7,386 (33.7%) on GA therapy and 14,343 patients with 36 months of follow-up, including 4,693 (32.7%) who were using GA. The declining size of the samples with each extension of follow-up is due to changes in insurance coverage that make patients ineligible for inclusion in the claims database.

Age and gender were available for all three patient groups. Patient characteristics for the overall cohorts and GA initiator cohorts are presented in Table 1. The overall sample and GA initiating patients were similar with respect to age and gender. The 12-, 24- and 36-month cohorts also had similar demographic characteristics. Approximately 50% of patients were between 36 and 50 years of age at the time of the first identified MS treatment in the study period. About three-quarters (76%) of the overall sample were female. The percentage of females in the GA initiator cohort was slightly higher at 78%.

**Table 1:** Patient demographic characteristics*

	**12 months**		**24 months**		**36 months**	
	**Overall**	**GA**	**Overall**	**GA**	**Overall**	**GA**
N	35,312	12,144	21,927	7,386	14,315	4,693
**Age**						
≤18	0.6%	0.7%	0.7%	0.7%	0.8%	0.8%
19-35	22.4%	21.7%	21.1%	20.5%	19.2%	18.8%
36-50	50.2%	50.3%	51.5%	51.7%	52.3%	52.4%
51-62	24.2%	24.5%	24.5%	24.6%	25.6%	25.7%
≥63	2.6%	2.8%	2.3%	2.5%	2.2%	2.2%
**Gender**						
Female	76.5%	78.2%	76.7%	78.6%	76.6%	78.6%

*Cohorts are subsets of one another.

### 12 Months following DMT initiation

**Figure 1:**
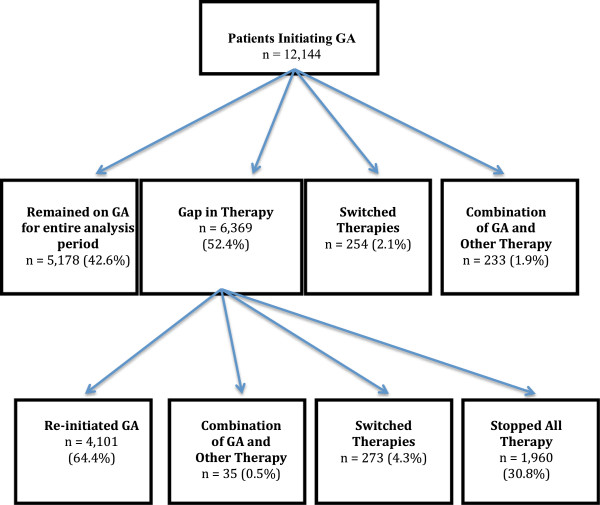
GA persistence experience at 12 months.

Of patients initiating GA, 42.6% (5,178) remained on GA for the entire 12 months without a gap in therapy of 15 days or more; 2.1% (254) switched from GA to another MS therapy; 1.9% (233) appeared to use GA as combination therapy, and 52.4% (6,369) had a gap in therapy of ≥15 days. Of the GA patients with a gap in therapy, 64.4% (4,101) re-initiated GA, 4.3% (273) switched to another therapy, and 30.8% (1,960) discontinued all DMT. Among patients with a minimum of 12 months of follow-up, the persistence rate with GA was 79.5%. When there was a regimen change, the average time to the first change in treatment was 124 days. The 12-month persistence experiences for those initiating GA are presented in Figure 1.

For the full DMT-treated cohort, 47.2% (16,665) of the overall cohort remained on their initial treatment for the entire year without a gap; 10.0% (3,454) switched to a different MS therapy. A gap in therapy of 15 days or more was observed for 17,222 (48.8%) of the overall cohort. Of the patients with a gap in therapy, 61.4% (10,573) re-initiated their initial therapy and 44.9% (7,727) discontinued all disease-modifying therapy (DMT). The persistence rate for the entire DMT cohort over 12 months was 68.3%. Of those using DMT for 12 months, more than ninety-five percent of patients were on monotherapy for their MS, while 4.6% of patients appeared to receive combination therapy at some point during the analysis period. For those with a change in therapy regimen, the average time to the first change in treatment was 127 days.

### 24 Months following DMT initiation

**Figure 2:**
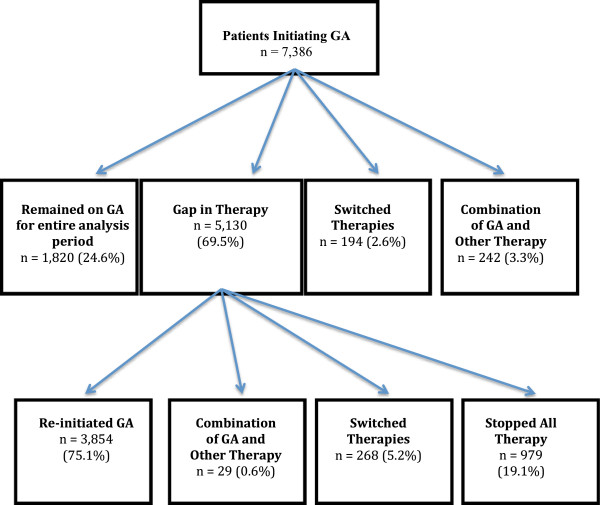
GA persistence experience at 24 months.

The persistence experience of GA patients over 24 months is outlined in Figure 2. Of the 7,386 patients initiating GA, 24.6% remained on GA for the 24 months without a gap in therapy of 15 days or more; 2.6% switched from GA to another therapy, and 3.3% of patients appeared to use GA as combination therapy. Nearly seventy percent of patients had a gap in therapy of 15 days or more. Of the patients whose first change of regimen was a gap, 75.1% reinitiated GA, 0.6% appeared to add another MS therapy (combination therapy); 5.2% switched to another therapy, and 19.1% discontinued DMT. Accounting for those who switched therapies or stopped using GA, the persistence rate with GA for those with a minimum of 24 months of follow-up was 80.5%.

For the 24-month cohort of GA users who experienced changes in their regimen, the average time to the first change in MS treatment was 222 days. The average time to the addition of a second therapy was 204 days, a first gap of 15 days or more occurred an average of 218 days into treatment; and a switch to another MS therapy occurred, on average, 227 days after beginning GA. After the initial therapy gap of 15 days or more, patients reinitiating GA did so at 54 days on average, with a treatment switch occurring an average of 109 days after the initial treatment gap.

In contrast, 30% (6,484) of the overall DMT-treated cohort remained on their initial therapy for the entire 24 months without a gap of 15 days or more; 16.0% (3,448) of patients switched to another MS therapy; and 67% (14,650) had a gap in therapy ≥15 days. Of the patients with a gap in therapy, 51.5% (11,172) re-initiated their initial MS therapy; 7.6% appeared to be on combination therapy at some point during the 24-month period; and 30% (6,658) discontinued all DMT. The persistence rate with any DMT over the 24 months was 53.9%. When there was a regimen change, the average time to the first change in therapy was 215 days.

### 36 Months following DMT initiation

**Figure 3:**
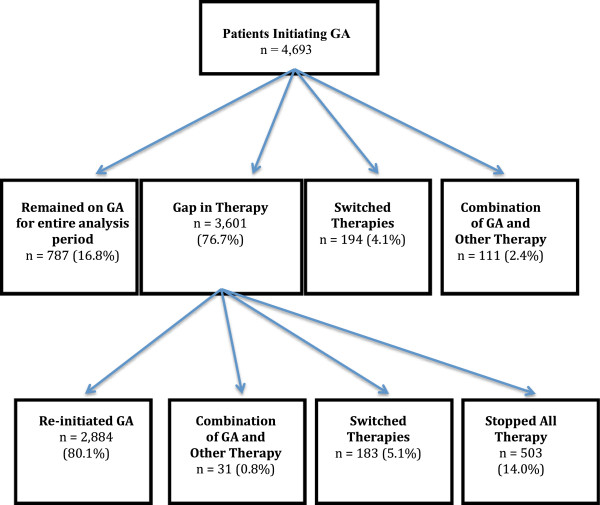
GA persistence experience at 36 months.

Figure 3 describes the persistence experience of GA patients at 36 months after initiation. Of those patients using GA, 16.8% (787) stayed on GA for the entire 36-month period without a gap in therapy of 15 days or more. 4.1% (194) switched from GA to another MS therapy and 2.4% (111) appeared to be using GA as combination therapy. Of patients with a gap in therapy of 15 days or more (3,601; 76.7%), 80.1% (2,884) reinitiated GA therapy; 5.1% (183) switched to another therapy and 14.0% (503) discontinued all therapies. Accounting for those who switched from GA to another therapy or discontinued GA and all DMT, the persistence rate with GA after 36 months of followed was 81.2%. When a regimen change was made, the average time to the first change in treatment was 296 days.

For the full DMT-treated cohort at 36 months after initiation of a DMT, 21.0% (2,961) of patients remained on their initial therapy; 20% (2,870) had switched therapies and 76.0% (10,912) had a gap in therapy ≥15 days. Of the patients with a gap in therapy, 56.0% (7,936) reinitiated their initial therapy. Combination therapy was observed in 9.9% of patients at some point in the analysis period and 14% (1,416) discontinued all DMT yielding a persistence rate with all available DMT of 70.1%. When a regimen change was made, the average time to the first change in treatment was 309 days.

## Discussion

Persistence for this investigation was defined as continuous use of the same DMT for the duration of follow-up (i.e., 12 months, 24 months, 36 months) regardless of treatment gaps. Among patients with a minimum of 12 months of follow-up, the persistence rate with GA was 79.5% while for the full cohort of DMT-treated patients the persistence rate at 12 months was 68.3%. This can be compared to the persistence investigation conducted by Reynolds et al. using a different US administrative claims database with MS patient-level data from 1996 to 2005 [25]. They found the persistence rate at 12 months for GA was 68.5%, and for all DMTs included in their study it was 65.1%. Differences in persistence rates between the studies may be the result of the time windows used for data extraction, and the sizes of the MS patient samples available for study. This study with a data extraction window of 2001 to 2010 included NZ that was not available for the Reynolds study. This could have resulted in more therapy switching and the corresponding washout periods without DMT that would decrease the persistence rates for all DMT. The data extraction time window used by Reynolds et al. (1996–2005) corresponds to the entry to the market of GA, and IFNβ-1a IM in 1996 and IFNβ-1a SC in 2002. Patients beginning new therapies were in the most vulnerable period for interruptions in therapy and that may be reflected in lower persistence rates than those seen in the later time window of this study. Reynolds et al. included 6,134 MS patients in their investigation, while this study had 35,312 MS patients available for the 12-month cohort. The characteristics of the patient samples in terms of age, gender and geographic distribution were very similar.

When patients with a minimum of two years of follow-up were examined for this study, the persistence rate for GA increased slightly to 80.5% and the corresponding rate for the full DMT-treated cohort fell to 53.9%. With 36 months of follow-up, the persistence rate for GA increased modestly to 81.2% and the persistence rate for the full DMT-treated cohort rose to 70.1%. Relative to the 12-month cohort, the persistence rates for GA remained stable for 24 and 36 months with modest increases in therapy switching and decreases in discontinuation. For the full DMT-treated cohort there was greater fluctuation in persistence rates over the three years. The proportion switching therapies increased with duration of follow-up while the proportion discontinuing DMT increased from 12 months to 24 months but declined after 36 months. This may reflect the introduction NZ in 2006 and the fact that patients needed to stop all DMT for several months for a washout period before they initiated NZ therapy. Results from this investigation do show some similarity to those of other investigators. Margolis et al. followed a cohort of newly diagnosed MS patients for an average of 35.7 ± 17.5 months; those who were treated with DMT had a persistence rate of 72.3% [26]. Rio et al. reported that 80.0% of their DMT users continued on the same therapy without interruption after a mean follow-up of 47 months [19], and Tremlett et al. reported that at the end of study follow-up (mean 2.4 years), the persistence rate for GA was 80.0% [27].

Gaps in MS therapy are commonplace and this study shows that they increase with the duration of all DMT. However, the proportion of GA users who re-initiated therapy following gaps in therapy increased over time from 64.4% at 12 months to 80.1% at 36 months. This was in contrast to the full DMT-treated cohort that maintained a therapy re-initiation rate of 50-60% over 36 months. The lower re-initiation rate may be attributed to some patients with more highly active disease who were hospitalized for a relapse, treated with corticosteroids, or taken off their DMT to begin another therapy. Regardless of DMT, the relatively high percentage of patients re-initiating the same therapy after a therapy gap may indicate that these patients are experiencing more short-term issues such as forgetfulness, failure to re-order medication, a problem with reimbursement or the desire to take a drug holiday with or without a physician’s approval.

The consequences of medication gaps have begun to be reported and gaps have been shown to be associated with an increased risk of MS relapse [27-29]. Tremlett et al. reported that therapy gaps were associated with a shorter time to first on-study relapse and trend towards future disease progression when compared to patients without missed doses [27].

Therapy regimen changes such as switching medication and stopping DMT interfere with persistence. In this study the rate of therapy switching increased over time; 4.3% of the GA cohort switched therapies in the first 12 months, 6.3% in 24 months and 8.0% over 36 months. This trend was consistent with the full DMT-treated cohort though the switching proportions for the GA cohort were somewhat lower than those for the overall sample: 10% over 12 months, 16% in 24 months and 20% over 36 months. This may reflect the results of Reynolds et al. who reported the lowest switch rates for GA relative to IFNβ [25]. When compared to other published results of therapy switching in MS, Margolis et al. reported more than twice as much DMT switching (21.3%) at 12 months as the 9.0% reported by Reynolds et al. [25,26]. and the 10.0% reported in this study. However, for GA users specifically, Reynolds et al. reported somewhat higher switch rates than those seen with this study: 3.9% had switched to another DMT in the first 6 months, 6.4% had switched over 12 months, and 8.3% switched over 18 months [25]. This may be related to the earlier window of data extraction used by Reynolds et al. corresponding to the market introduction of GA.

Unlike therapy switching that increased with duration of therapy, the proportion discontinuing declined with duration of therapy in this study. This is consistent with the findings of others who have noted that the most vulnerable time for therapy discontinuation is the first six months of therapy [18,23]. There is evidence to suggest that the timing of therapy discontinuation is earlier when the reason is an adverse drug event and later when the reason for stopping is a perceived lack of therapy effect [18,30]. Several investigators have explored the reasons for therapy discontinuation and they include factors related to the disease such as the type of MS, the level of disability or physician-documented disease progression, adverse effects, patients’ perceptions of therapy ineffectiveness, the presence of cognitive dysfunction, and/or depression and therapy cost [14-21].

## Limitations

Claims database analysis allows for estimation of real-world treatment patterns, including persistence with individual therapies, and the strength of our analysis derives from the large, geographically diverse population studied. All retrospective database analyses are subject to certain limitations, and the results of this study must be interpreted with appropriate consideration of these limitations. Claims data are collected primarily for payment purposes, not research, and are subject to coding errors. Presence of a diagnosis code for MS on a medical claim is not positive presence of MS. The presence of a claim for a filled prescription does not necessarily indicate that the medication was consumed or that it was taken as prescribed. Medications filled over-the-counter or provided as samples by the physician were not observed in the claims data, and regimen changes were based on filled prescriptions. Claims-based data are constrained by coverage limitations that determine the data available and limit generalizability of results to managed care patients.

Limitations specific to this study include the inability to ascertain the reasons why patients had a change in regimen. Although claims data do not provide this information, key factors influencing persistence and discontinuation in MS patients in the literature include those mentioned previously. Additionally, the study window of 2001 to 2010 did not allow an investigation of persistence with the new generation of oral therapies for MS.

## Conclusions

Persistence with GA was 80% over the 12-month, 24-month and 36-month time periods. Although treatment gaps of 15 days or more were common for all DMT, higher rates of therapy re-initiation following treatment gaps was seen with GA than with the full DMT-treated cohort and this contributed to consistently high persistence rates with GA. Persistence with the full DMT-treated cohort fluctuated and never exceeded 70.0% over the three time periods. Rates of switching to another MS therapy were lower for patients initiating GA than for the full cohort of DMT-treated patients. Therapy discontinuation declined for all DMT over longer periods of follow-up. Persistence with MS therapy is essential to achieve the desired outcomes in MS. With the more recent introduction of several oral DMTs, further investigation of long-term therapy persistence in MS should be pursued.

## Abbreviations

DF: Dimethyl fumarate; DMT: Disease-modifying therapy; FG: Fingolimod; GA: Glatiramer acetate; ICD-9: International classification of diseases, Ninth Revision; IFNβ-1a IM: Intramuscular interferon beta-1a; IFNβ-1a SC: Subcutaneous interferon beta-1a; IFNβ-1b: Interferon beta-1b; MS: Multiple sclerosis; NDC: National drug codes; NZ: Natalizumab; RRMS: Relapsing-remitting multiple sclerosis; TF: Teriflunomide; UHG: United health group; US: United States.

## Competing interests

MOB was an employee of Teva Pharmaceuticals, the distributor of glatiramer acetate, when this study was conducted and has received consulting fees for editing this manuscript. AC and ES are employees of OptumInsight that conducted the research under contract with Teva Pharmaceuticals. Support for this manuscript was provided by Teva Pharmaceuticals.

## Authors’ contributions

MOB conceived of the study, participated in the design of the study and interpretation of data, and helped to draft the manuscript. AC participated in the design of the study, interpretation of data and recommended revisions to the manuscript. ES participated in the design of the study, performed the data analysis, and helped with interpretation of data and drafting of the manuscript. All authors read and approved the final manuscript.

## Pre-publication history

The pre-publication history for this paper can be accessed here:

http://www.biomedcentral.com/1471-2377/14/11/prepub
